# Construction of Pseudomolecule Sequences of the *aus* Rice Cultivar Kasalath for Comparative Genomics of Asian Cultivated Rice

**DOI:** 10.1093/dnares/dsu006

**Published:** 2014-02-26

**Authors:** Hiroaki Sakai, Hiroyuki Kanamori, Yuko Arai-Kichise, Mari Shibata-Hatta, Kaworu Ebana, Youko Oono, Kanako Kurita, Hiroko Fujisawa, Satoshi Katagiri, Yoshiyuki Mukai, Masao Hamada, Takeshi Itoh, Takashi Matsumoto, Yuichi Katayose, Kyo Wakasa, Masahiro Yano, Jianzhong Wu

**Affiliations:** 1 Agrogenomics Research Center, National Institute of Agrobiological Sciences, 2-1-2 Kannondai, Tsukuba, Ibaraki 305-8602, Japan; 2 Genome Research Center, NODAI Research Institute, Tokyo University of Agriculture, 1-1-1 Sakuragaoka, Setagaya, Tokyo 156-8502, Japan; 3 Department of Bioscience, Faculty of Applied Bioscience, Tokyo University of Agriculture, 1-1-1 Sakuragaoka, Setagaya, Tokyo 156-8502, Japan

**Keywords:** *Oryza sativa*, genome re-sequencing, comparative genomics, SNPs and indels, gain and loss of genes

## Abstract

Having a deep genetic structure evolved during its domestication and adaptation, the Asian cultivated rice (*Oryza sativa*) displays considerable physiological and morphological variations. Here, we describe deep whole-genome sequencing of the *aus* rice cultivar Kasalath by using the advanced next-generation sequencing (NGS) technologies to gain a better understanding of the sequence and structural changes among highly differentiated cultivars. The *de novo* assembled Kasalath sequences represented 91.1% (330.55 Mb) of the genome and contained 35 139 expressed loci annotated by RNA-Seq analysis. We detected 2 787 250 single-nucleotide polymorphisms (SNPs) and 7393 large insertion/deletion (indel) sites (>100 bp) between Kasalath and Nipponbare, and 2 216 251 SNPs and 3780 large indels between Kasalath and 93-11. Extensive comparison of the gene contents among these cultivars revealed similar rates of gene gain and loss. We detected at least 7.39 Mb of inserted sequences and 40.75 Mb of unmapped sequences in the Kasalath genome in comparison with the Nipponbare reference genome. Mapping of the publicly available NGS short reads from 50 rice accessions proved the necessity and the value of using the Kasalath whole-genome sequence as an additional reference to capture the sequence polymorphisms that cannot be discovered by using the Nipponbare sequence alone.

## 1. Introduction

Over the last decade, technological developments have led to the generation of an unprecedented amount of genomic data for model organisms, providing basis for the discovery of their genes and understanding their genetics. The sequence of the first plant genome, from the dicot *Arabidopsis thaliana*, was completed and published at the end of 2000.^1^ This sequence has served as a common reference for gene annotation and comparative genomics.^2,3^ In particular, using the whole-genome sequence, information provided by the next-generation sequencing (NGS) technologies (the new data are emerging from the 1001 Genomes Project launched in early 2008; http://1001genomes.org/) has dramatically increased the numbers of known genetic variants [up to several millions of single-nucleotide polymorphisms (SNPs)] in this model plant. The monocot species Asian rice (*Oryza sativa* L.) is one of the most important cereal crops, feeding more than half of the global population, especially in Asian countries. The International Rice Genome Sequencing Project (IRGSP) deciphered the whole genome of the subspecies *japonica* cultivar Nipponbare in 2005, and released a map-based high-quality sequence covering >95% of its genome.^4^ This sequence has provided a foundation for our understanding of rice genome organization, including both genes and repetitive sequences, and accelerated functional genomic studies in rice. To date, ∼700 rice genes controlling various morphological and physiological traits, including resistance to biotic and abiotic stresses, have been functionally characterized.^5^ With the Nipponbare sequence as a reference, genome re-sequencing of a large number of rice accessions has led to the discovery of millions of SNPs and insertion/deletion sites (indels), enabling genome-wide association studies (GWAS) aimed at identifying agronomically important genes in rice.^6,7^

To meet the challenges deriving from rapid population growth and worldwide climate change, continuous efforts to increase rice production by using the genetic improvement technologies will be of great importance. One of the world's oldest crops (domesticated ∼10 000 years ago), rice is traditionally classified into two major subspecies, *indica* and *japonica*.^8–10^ Owing to the deep genetic structure of rice evolved during domestication and adaptation and its autogamous breeding system, current *O. sativa* cultivars and landraces can be subdivided in more detail into five genetically differentiated groups: *indica*, *aus*, *aromatic*, *temperate japonica*, and *tropical japonica*.^11^ While the reference Nipponbare sequence is particularly useful for evolutionary and functional studies, its use for extensive analysis of genome diversity remains limited because of considerable inter- and intra-species and even intra-subspecies chromosomal rearrangements, such as insertions and deletions, duplications, inversions, translocations, and transpositions.^12–15^ Consistent with the above observations, the portion of uniquely mapped reads among the NGS short-read sequences from 50 cultivated and wild rice accessions against the Nipponbare reference genome varied greatly, from 73.0 to 93.0%, with the highest rate in *temperate japonica* accessions followed by *tropical japonica*, *aromatic*, *aus*, *indica*, and wild rice accessions.^7^ The power of GWAS for identifying rice genes depends greatly on the number and quality (high accuracy and even distribution along each rice chromosome) of SNPs, particularly when the analysis is conducted with germplasms collected within a subspecies or local populations.^16,17^ Moreover, the absence of some genes conferring tolerance to submergence or phosphorus deficiency from the Nipponbare genome caused by DNA insertions or deletions has been reported, strongly indicating that a single-reference genome is insufficient for discovery of novel genes or comprehensive transcriptome analysis through the RNA-Seq technology in rice.^18–20^ Because of the deep genetic structure in *O. sativa*, thereafter, new reference sequences from additional rice cultivars are needed, although chromosomal mapping and *de novo* assembly of the NGS reads are still challenging.^21,22^

Rice cultivar Kasalath belongs to the *indica* subspecies or *aus* group of *O. sativa*, which has higher genome diversity than the *japonica* subspecies.^11^ Carrying a number of beneficial traits such as early maturity and tolerance to drought and phosphate deficiency, this cultivar, together with Nipponbare, has been particularly useful for developing a series of important genetic and genomic resources that have already contributed greatly to the molecular and functional analysis of rice chromosomes.^12,18,23–29^ In this study, we sequenced the whole genome of Kasalath rice by using two NGS platforms, Illumina (GAIIx and HiSeq2000) for short reads and Roche 454 (GS FLX Titanium and GS FLX+ Titanium) for long reads. We performed *de novo* assembly and chromosomal mapping of the NGS read sequences. In addition, we carried out the transcriptome analysis with RNA-Seq data obtained from young leaves and panicles of Kasalath by using the GAIIx for annotation of expressed sequences. Comparative analysis of the Kasalath sequence and those of other rice cultivars confirmed its value as a new reference genome to facilitate future evolutionary and functional genomic studies in rice.

## 2. Materials and methods

### 2.1. Library construction and genome re-sequencing

Total genomic DNA of Kasalath was extracted from young leaves of a single plant by using the cetyltrimethylammonium bromide method.^30^ We constructed DNA libraries with insert sizes of 800–1500 bp according to standard manufacturer's protocols (http://www.454.com/; Basel, Switzerland) to generate long-read sequences by using Roche 454 pyrosequencing technology (GS-FLX Titanium and GS-FLX+ platforms) as described previously.^31^ We also constructed libraries with insert sizes of 250–400 bp according to the manufacturer's instructions (Illumina, San Diego, CA, USA) to produce short single or paired-end reads on the Illumina GAIIx or HiSeq 2000 platforms.^31^ To facilitate annotation of the expressed sequences, we constructed cDNA libraries with insert sizes of 350–400 bp from total RNA samples prepared from the young leaves or young panicles of Kasalath, and used these libraries to generate short-read RNA-Seq data on the Illumina GAIIx instrument as described.^32^

### 2.2. Genome assembly

Raw sequence read data generated on both platforms were preprocessed to trim low-quality or adapter sequences on both ends as described previously.^31^ Sequencing errors in the Illumina data were corrected by String Graph Assembler (SGA) software v. 0.0.20 with ‘*k*-mer = 55’.^33^

To construct the Kasalath pseudomolecules (Supplementary Fig. S1), we first performed *de novo* assembly of 454 reads into sequence contigs by using Celera Assembler v. 7.0 software with utgErrorRate = 0.015, ovlErrorRate = 0.03, cnsErrorRate = 0.05, cgwErrorRate = 0.05, utgGraphErrorRate = 0.015, utgMergeErrorRate = 0.02, and default values for other options. To improve sequence accuracy, we then mapped the error-corrected Illumina reads to the above contigs by Burrows–Wheeler Alignment (BWA) v. 0.6.2 software with the ‘-e 10’ option.^34^ With the mapped paired-end reads, we further refined the alignments around the indel sites by using Genome Analysis Toolkit (GATK)^35^ software and discarded the putative polymerase chain reaction duplicates by using Picard software (http://picard.sourceforge.net/). Errors in each sequence contig were detected by calling variants using the SAMtools mpileup function with ‘-q 20 -Q 20’ options.^36^ Errors were corrected if the detected variants were homozygous with a quality score of ≥30, sequencing depth of ≥10, and frequency of ≥70%. This error correction procedure was performed twice to ensure sequencing accuracy. After again mapping the error-corrected Illumina reads to the error-corrected 454 contigs, we finally conducted a hybrid *de novo* assembly by merging the error-corrected 454 contigs with the unmapped Illumina reads by using SGA with ‘-m 75 -d 0.4 -g 0.1 -r 30’ options.

### 2.3. Generation of Kasalath pseudomolecules

All contigs of ≥500 bp were subjected to chromosomal mapping. First, we physically mapped their sequences to the Nipponbare reference genome (IRGSP 1.0)^31^ by using MUMmer v. 3.23 software (NUCmer) with default settings.^37^ We selected the optimal alignments by using delta-filter commands; the coordinates of each contig were displayed by using the show-coords command.^37^ All aligned contigs with values below the thresholds (90% nucleotide identity and 80% sequence coverage) were removed. If a contig was split into two or more fragments, we considered that it might correspond to genomic sites with large indels relative to the Nipponbare sequence. To determine the insertion sites, we used a fixed threshold of unaligned fragments of ≥100 bp with flanking sequences of ≥200 bp (Supplementary Fig. S2A). To determine the deletion sites, we used gapped alignments of 100–50 000 bp with flanking sequences of ≥200 bp (Supplementary Fig. S2B).^38^

Bacterial artificial chromosome (BAC)-end sequences (BESs) from Kasalath were used to anchor the sequence contigs that could not be aligned to the Nipponbare genome by MUMmer. We mapped all Kasalath BESs (DDBJ accessions AG831174–AG909573; http://rgp.dna.affrc.go.jp/E/publicdata/kasalathendmap/index.html) onto the Kasalath contigs by BLASTN algorithm with ‘*e*-value 1.0*e*^−5^’ option.^39^ We selected the best positions with ≥90% nucleotide identity and ≥95% sequence coverage, and used only the uniquely aligned BESs for further analysis. We also mapped the Kasalath BESs to the Nipponbare genome sequence by selecting the best positions with ≥90% nucleotide identity and ≥90% sequence coverage, and retained only the pairs of BESs uniquely mapped at a distance of <300 kb on the Nipponbare genome. Unmapped Kasalath contigs were anchored onto the Nipponbare genome if they (i) contained uniquely aligned BESs mapped onto a Nipponbare genomic region where no Kasalath contigs have been assigned by MUMmer and (ii) had the mates of BESs aligned on a different contig already mapped on the Nipponbare sequence by MUMmer. Finally, we used the Illumina paired-end sequences to anchor the remaining Kasalath contigs to the Nipponbare sequence in the same manner as for the construction of chromosome pseudomolecules.

### 2.4. Transcriptome analysis

The RNA-Seq reads of Kasalath were mapped onto its pseudomolecules by Tophat v. 2.0.8b software with the ‘-min-intron-length 67 --max-intron-length 3608’ options.^40^ The thresholds for the intron length corresponded to the 1st and 99th percentiles of the distribution of intron length, as retrieved from the annotations in the Rice Annotation Project (RAP) database.^41^ In addition, we set the ‘−G’ option on the basis of the intron/exon structures in the pseudomolecules converted from the Nipponbare genome annotated by the RAP. Gene structures predicted by Cufflinks v. 2.1.1 software individually for the young leaves and young panicles were merged by Cuffmerge software.^42^ DNA sequences of predicted transcripts were mapped onto the Nipponbare genome or proteome^41^ sequences by the BLASTN algorithm and est2genome tool^43,44^ with thresholds of ≥90% nucleotide identity and ≥70% sequence coverage.

### 2.5. Detection of SNPs and indels among rice cultivars

Pseudomolecule sequences were compared among *japonica* rice Nipponbare (IRGSP 1.0, http://rapdb.dna.affrc.go.jp/), *indica* rice 93-11 (http://rise2.genomics.org.cn/page/rice/index.jsp), and *aus* rice Kasalath by using the MUMmer program to detect the existence of SNPs and indels. To ensure that large indels (≥100 bp) between any two cultivars were not due to misassembled contigs, we mapped all Illumina reads of the Kasalath genome to its pseudomolecule sequences by using BWA to confirm that the boundaries of the insertions (Supplementary Fig. S2A) and deletions (Supplementary Fig. S2B) were covered by at least five overlapping reads stepped over by their paired sequences. Each SNP and indel was annotated by SnpEff (http://snpeff.sourceforge.net/index.html) to predict the effects of variants on genes.

### 2.6. Chromosomal mapping of the publicly available short-read sequences by using multiple rice pseudomolecules

Publicly available sequence data generated by the Illumina GAII instruments from 50 accessions of cultivated and wild rice at ∼15× coverage were downloaded from the NCBI Short Read Archive (accession number SRA023116).^7^ By using BWA,^34^ we aligned these sequences to the pseudomolecule sequences of Nipponbare, Kasalath, and 93-11 to examine the efficiency of chromosomal mapping. By using TASUKE, a web-based application developed recently for visualization of large-scale re-sequencing data,^45^ we constructed a genome viewer to display the sequences of and the structural variations among the above rice accessions with reference to the genomic sequence of Kasalath instead of Nipponbare.

## 3. Results and discussion

### 3.1. Kasalath pseudomolecules constructed from 330.55 Mb of sequences

By performing *de novo* assembly of 2.49 Gb of sequences (>6× coverage) generated by Roche 454 (1.73 Gb from GS-FLX Titanium with an average read length of 386.1 bp, and 0.76 Gb from GS-FLX+ with an average read length of 593.8 bp) with Celera Assembler, we created 109 362 contigs containing 296.3 Mb with an N50 length (minimum length of contigs representing 50% of the assembly) of 3.2 kb. To increase coverage and accuracy of genomic sequences, we additionally generated a total of 57.47 Gb of Kasalath sequences (>148× coverage) by using Illumina GAIIx and HiSeq2000. On the basis of trimmed and error-corrected Illumina sequences, we corrected the sequencing errors within all contigs initially assembled from the Roche 454 reads. Finally, we conducted the hybrid *de novo* assembly by using all of the above-sequence data from both Illumina and Roche 454, which produced 51 550 contigs containing a total of 330.55 Mb non-overlapping sequences, which corresponds to 88.6% of the published Nipponbare sequence (373.25 Mb) (Table 1). Approximately 72% (36 932) of these Kasalath contigs, corresponding to 87.7% (289.80 Mb) of the total assembled sequences, were successfully anchored to the 12 chromosomes (Supplementary Table S1), covering 292.49 Mb of the Nipponbare reference genome. The total length (35 914 803 bp) of Kasalath contigs anchored on chromosome 1 by the hybrid *de novo* assembly was longer than that (32 835 386 bp) achieved only by single-reference (Nipponbare) mapping using BWA (details not shown). By using the MUMmer alignment software, we mapped the above two sequences to a BAC-based genomic sequence (41.37 Mb) of Kasalath chromosome 1^12^ (≥99% nucleotide identity), which revealed chromosome coverage of 82.3 and 73.0%, respectively. Therefore, our assembly of NGS reads can link genomic sequences to the Kasalath chromosomal regions, which is not possible by using reference mapping only. Such chromosomal regions could be cultivar-specific; these differences may be caused by the insertions or deletions of DNA segments, and in some cases they might be of great importance for the maintenance and use of rice genetic resources. For example, recent cloning and functional analysis of a major quantitative trait locus for phosphorus-deficiency tolerance (*Pup1*) in rice placed this gene within a chromosomal region of ∼90 kb on Kasalath chromosome 11, which was absent in the Nipponbare genome.^18^ In our present study, a total of 18 contigs (60.25 kb) were successfully assembled from the above genomic region of Kasalath, which fully covered the *Pup1*-specific protein kinase gene (*PSTOL1*).

**Table 1.** Statistics of *de novo* assembly and chromosomal mapping of Kasalath NGS reads to Nipponbare pseudomolecules

	No. of contigs	N50 (bp)	Maximum L (bp)	Mean L (bp)	Total L (bp)
Mapped	36 936	13 728	103 131	7847	289 796 664
Unmapped	14 822	3615	43 777	2749	40 748 813

L, length; N50, minimum length of contigs representing 50% of the assembly.

### 3.2. Genome-wide diversity among Kasalath, Nipponbare, and 93-11

Sequence alignment by MUMmer revealed SNPs at 2 787 250 nucleotide sites between Kasalath and Nipponbare (alignment length of 278.75 Mb) and 2 216 251 nucleotide sites between Kasalath and 93-11 (alignment length of 259.00 Mb) (Table 2). Thus, the SNP frequency was 1.00% between *aus* and *japonica* and 0.86% between *aus* and *indica*, consistent with the genetic structure of *O. sativa* reported so far.^8–11^ Kasalath and 93-11 shared 1 378 591 common SNPs in comparison with the Nipponbare genome, which provides useful genomic resources for future studies of domestication and subspeciation of Asian cultivated rice. The SNPs present only between Kasalath and 93-11, two closely related cultivars, offer great potential for the discovery of naturally occurring mutations that might be associated with recent phenotypic changes that appeared during local adaptation after the divergence of *japonica* and *indica*.

**Table 2.** Statistics of SNPs and indels detected between Kasalath, Nipponbare, and 93-11 genomes

	Kasalath–Nipponbare		Kasalath–93-11	
	SNPs	Large indels	SNPs	Large indels
chr01	318 375	921	252 557	506
chr02	253 524	651	184 485	383
chr03	278 482	749	204 712	405
chr04	233 426	617	212 291	329
chr05	244 149	616	179 973	294
chr06	241 274	648	210 155	373
chr07	231 566	605	155 910	300
chr08	214 710	535	166 666	255
chr09	179 050	434	141 896	232
chr10	193 491	531	149 212	241
chr11	211 931	533	185 340	195
chr12	187 272	553	173 054	267
Total	2 787 250	7 393	2 216 251	3780

**Figure 1.**
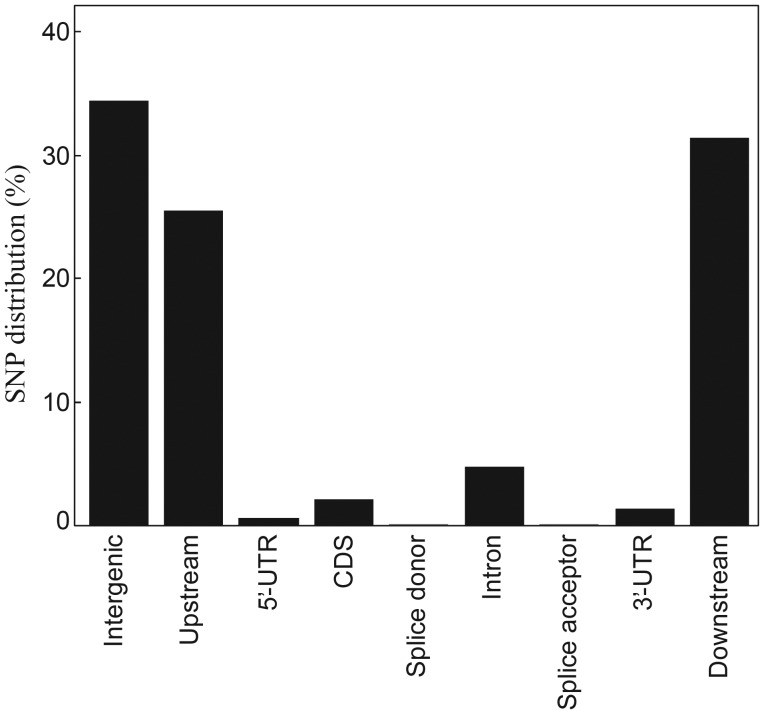
Distribution pattern of SNPs detected between the genomes of Nipponbare and Kasalath cultivars.

This genomic information should help to explain in-depth the molecular mechanisms underlying not only the evolution, but also the functions of rice genomes. A total of 37 869 genes have been annotated in the Nipponbare genome.^41^ We found that most of the SNPs resided in non-genic regions. Only 5.1% (142 366) of the total SNPs detected between Nipponbare and Kasalath were located within protein-coding regions (Fig. 1). SNPs creating premature stop codons (nonsense mutations) or altering splice-site motifs can be expected to cause harmful effects on gene and protein function and eventually loss of function. We examined SNP presence and locations within 26 132 genes with sequences fully aligned between the Nipponbare and Kasalath genomes, and discovered that 902 genes had premature stop codons or splice-site motifs altered; of these, 33 seemed to have been pseudogenized in Kasalath. To compare the expression of the genes with and without harmful SNPs in the two cultivars, we carried out whole-transcriptome analysis in Nipponbare and Kasalath by using the RNA-Seq data for young leaves and young panicles. The fraction of genes specifically expressed in Nipponbare was significantly higher among the genes carrying harmful SNPs than among the genes without such SNPs (*P* < 10^−9^), suggesting that genes with harmful mutations are subject to pseudogenization.

**Figure 2.**
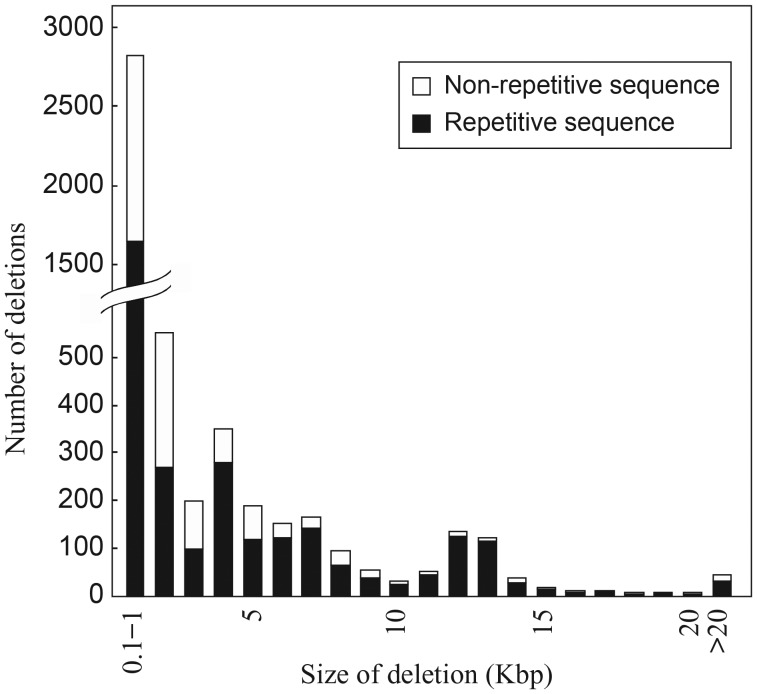
Size distribution and sequence classification of large deletions in Kasalath in comparison with Nipponbare.

Furthermore, we detected large indels at 7393 genomic sites between Kasalath and Nipponbare (100–38 041 bp; average length 1999 bp) and at 3780 genomic sites between Kasalath and 93-11 (100–15 333 bp; average length 735 bp) (Table 2), corresponding to large indel frequency of 0.003% (*aus*–*japonica*) and 0.001% (*aus*–*indica*). The total amount of indel nucleotides (completed sequences) in Kasalath relative to Nipponbare was 14.78 Mb (5026 deletions, 13.49 Mb; 2367 insertions, 1.29 Mb); much fewer indel sites were observed in Kasalath relative to 93-11 (2244 deletions, 1.84 Mb; 1,536 insertions, 0.94 Mb). We detected many more chromosomal sites for deletions than for insertions, probably owing to inefficiencies of *de novo* assembly of NGS short reads and chromosomal mapping of assembled contigs, especially for the genomic regions carrying recently duplicated segments or highly repetitive sequences. When we took into account the insertions containing partially assembled sequences, the total length of inserted sequences in Kasalath increased to 7.39 Mb, which, however, was still much less than that of the deleted sequences (13.49 Mb). These findings imply that the genome of Kasalath (estimated size of 363 Mb) is slightly smaller than that of Nipponbare (384–387 Mb).^31^ The distribution pattern of deletion sizes in Kasalath against Nipponbare displayed two peaks if deletions of <1 kb were ignored (Fig. 2 and Supplementary Fig. S2). The first and the largest peak appeared at 4 kb (3–5 kb), in which 58.5% of nucleotides were from repetitive sequences. The second peak was at 12–13 kb (11–14 kb), in which up to 62.7% of the nucleotides were from repetitive sequences. These data reveal the involvement of transposable elements, particularly those from the long-terminal-repeat retrotransposon families.^15^

### 3.3. Gain and loss of genes in Kasalath, Nipponbare, and 93-11

**Figure 3.**
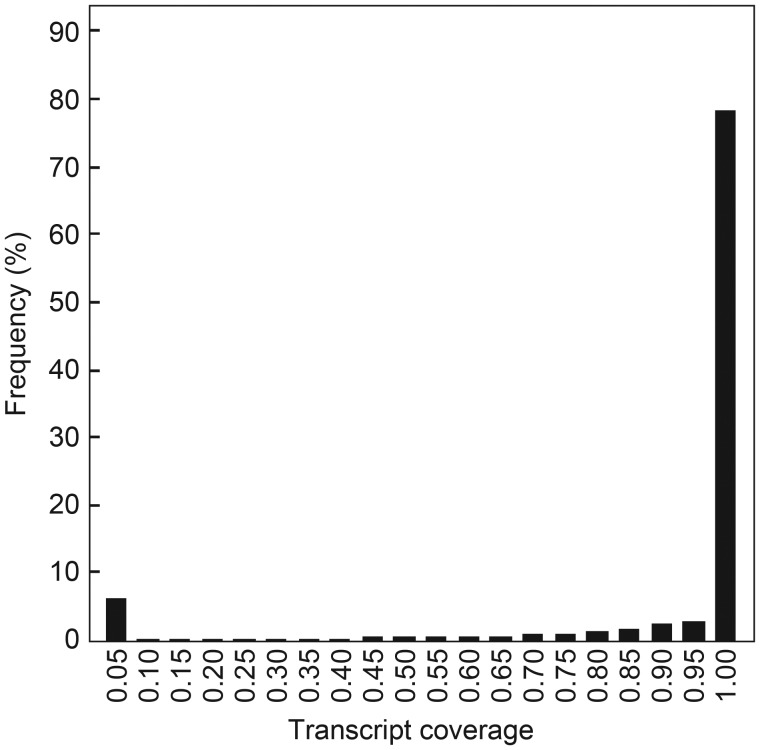
Nipponbare transcripts covered by Kasalath pseudomolecule sequences. The horizontal axis represents the sequence coverage (×100%) of each gene annotated on Nipponbare pseudomolecules.

About 72.0% of the Nipponbare chromosomal sites (67.6 Mb) uncovered by Kasalath pseudomolecules were masked as repetitive sequences. Clearly, up to 89.0% of the transcript sequences of Nipponbare were rescued by the assembled contigs of Kasalath (Fig. 3). This result indicates that most of the genic regions in Kasalath were captured through our re-sequencing and genome assembly. However, we still found that 6.3% (2828) of the total transcripts (44 536, including alternative variants) annotated in Nipponbare were likely absent in Kasalath (exon coverage <5%). To clarify whether these missing transcripts represented real changes of gene content between these cultivars, we examined the gene coverage by aligning the 93-11 genome (*indica*) to the Nipponbare reference genome. Interestingly, a similar number of the Nipponbare transcripts (2904) were likely missing in the 93-11 genome, of which 1278 were also absent in Kasalath (*aus*). These results clearly indicate that at least 3.1% of the genes in the *japonica* cultivar Nipponbare (1174 of 37 869 genes, excluding alternative variants) lack orthologs in *indica* and *aus* cultivars, mainly because of insertions or deletions. The frequency of the Nipponbare genes absent in Kasalath or 93-11 seemed to vary among the 12 chromosomes; chromosome 3 had the lowest value of 7.6 genes absent per Mb (Supplementary Fig. S3). As expected, an extremely high frequency (21.6 genes absent per Mb) was observed on chromosomes 11 and 12, which are characterized by recent generation of gene copies by tandem gene amplification and segmental duplication in the Nipponbare genome.^46^ Since these two chromosomes are known to carry the genes for agronomically important traits (such as resistance to blast, bacterial blight, viruses, and insects; photoperiod-sensitive male sterility; and salt tolerance),^47^ our comparison of the genomic sequences of different rice cultivars should provide fundamental information useful for our understanding of the evolution and function of these genes to the benefit of future molecular breeding programmes.

We obtained 2.3 Gb of the RNA-Seq reads from young leaves of Kasalath and 2.9 Gb from young panicles. This enabled us to perform whole-transcriptome analysis of the *aus* rice genome. By mapping these two datasets to Kasalath pseudomolecule sequences (all assembled sequences), we annotated 55 188 transcripts comprising 35 139 loci (Supplementary Fig. S4). To estimate the gain of genes in *aus* rice in comparison with *japonica* rice, we aligned all Kasalath transcript sequences to the Nipponbare pseudomolecules or protein sequences from the proteome database.^41^ A total of 2664 transcripts remained unmapped (<90% nucleotide identity and <70% sequence coverage); of these, 1226 unique to Kasalath (<50% sequence coverage). Of the 1226 transcripts, the translated sequences of 789 matched 535 known proteins. Analysis of the functional protein domains encoded by these transcripts revealed that protein kinases and disease resistance-related proteins were over-represented (Table 3), supporting the previous results of comparative genome analysis of Asian cultivated rice.^48^

**Table 3.** Top 10 over-represented functional domains in the genes found in Kasalath but not in Nipponbare

IPR000719	Protein kinase, catalytic domain
IPR000767	Disease resistance protein
IPR001245	Serine-threonine/tyrosine-protein kinase catalytic domain
IPR001611	Leucine-rich repeat
IPR002182	NB-ARC
IPR008271	Serine/threonine-protein kinase, active site
IPR011009	Protein kinase-like domain
IPR013210	Leucine-rich repeat-containing N-terminal, type 2
IPR013320	Concanavalin A-like lectin/glucanase, subgroup
IPR017441	Protein kinase, ATP-binding site

### 3.4. Chromosomal mapping of publicly available NGS short reads from 50 rice accessions to multiple reference sequences

**Figure 4.**
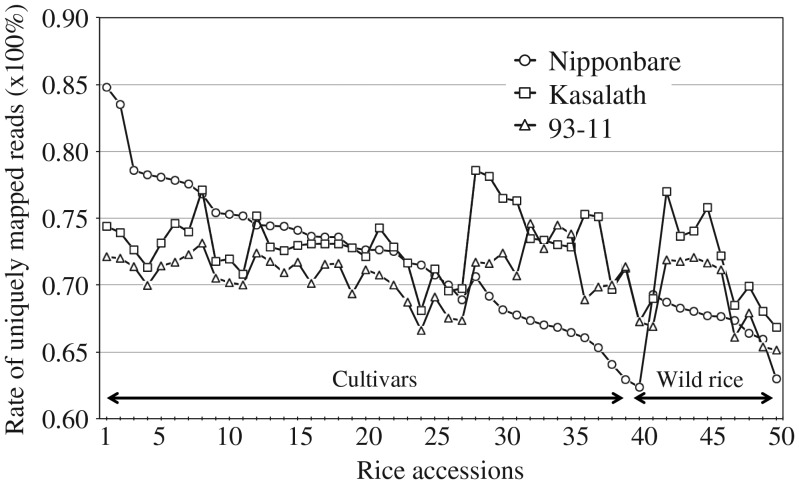
Rate of uniquely mapped NGS reads from 50 rice accessions by using Nipponbare, Kasalath, and 93-11 pseudomolecule sequences as references. Arabic numerals under the horizontal axis represent different accessions of cultivated and wild rice (see Supplementary Table S2 for details).

The map-based, high-quality sequence of Nipponbare has been typically used as a reference for not only comparative, but also functional genomics.^6,7,49–51^ In the present study, comparative analysis of genomic sequences of Kasalath, Nipponbare, and 93-11 led to the discovery of cultivar-specific sequences; some were associated with genes of agronomic importance such as *Pup1* in the Kasalath genome. About 7.39 Mb of inserted sequences were detected in Kasalath relative to Nipponbare, and 40.75 Mb of Kasalath sequences still remained unmapped to its chromosomes. This result emphasizes the necessity and importance of using pseudomolecule sequences as additional references for comparative genomic studies in rice to understand comprehensively its genome diversity, particularly among the cultivars of the *indica* subspecies and *aus*-type cultivars. We mapped the publicly available Illumina short reads derived from 50 diverse landraces and wild rice accessions^7^ to the pseudomolecule sequences of Kasalath, Nipponbare, and 93-11 (Supplementary Table S2). The mapping rate of unique reads (uniquely mapped reads/total reads × 100%) varied widely between the accessions, from 62.3 to 84.8% (Fig. 4). As expected, more sequence reads from the *aus* and *indica* varieties were mapped to the Kasalath and 93-11 genomes than to the Nipponbare genome, except for one *tropical japonica* accession (IRGC43397), which might have been previously misgrouped by phylogenetic analysis or its genomic DNA used for genotyping and sequencing was mislabelled. On the other hand, the mapping rates were low for all accessions when the 93-11 pseudomolecule sequence was used as a reference. This result indicates certain limitations in using the current 93-11 sequence for extensive comparative genomic studies in rice, probably because of its lower accuracy or poorer quality of sequence assembly than those of the Nipponbare and Kasalath pseudomolecules. A recent study has been performed to improve sequence quality and chromosome coverage by re-sequencing the 93-11 genome up to 36-fold depth.^49^ Detailed data on the gene annotation and the sequence and structural variations among the 50 rice accessions obtained in the present study by using the Kasalath pseudomolecules as a reference are accessible through our genome viewer (http://rice50ks.dna.affrc.go.jp/) developed on the basis of the TASUKE program (Supplementary Fig. S4).^45^

### 3.5. Conclusions

In this study, we performed deep sequencing (>154-fold coverage) by using NGS technologies and *de novo* assembly of the whole genome of the *aus* rice cultivar ‘Kasalath’. The assembled sequences cover 91.1% of the whole genome and 89.0% of the transcribed regions annotated on the basis of the reference Nipponbare genome. Besides millions of SNPs, comparative genomics revealed genome-wide sequence and structural variations, including thousands of large indels associated with the gain or loss of genes, between *japonica*, *indica*, and *aus*-type rice cultivars. Chromosomal mapping of the publicly available NGS reads from 50 rice accessions to Kasalath pseudomolecules demonstrated that its genomic sequence should be extremely useful as a new reference for future comparative genomic studies, particularly for capturing the sequence polymorphisms that could not be obtained by using the Nipponbare pseudomolecule sequences alone.

## Accession numbers

The genomic and RNA-seq sequences of Kasalath rice reported in this paper have been deposited in the DDBJ database with accession numbers DRA000968 and DRA001099.

## Supplementary data

Supplementary data are available at www.dnaresearch.oxfordjournals.org.

## Funding

This work was supported by grants from the Ministry of Agriculture, Forestry and Fisheries of Japan (Genomics for Agricultural Innovation, QTL5003 and GIR1001; Development of Genome Information Database System for Innovation of Crop and Livestock Production) and from the MEXT-Supported Program for the Strategic Research Foundation at Private Universities (S0801025).

## Supplementary Material

Supplementary Data
