# Supplementary material for: Construction of Pseudomolecule Sequences of the aus Rice Cultivar Kasalath for Comparative Genomics of Asian Cultivated Rice
Source: DNA Res. 2014 Feb 26;21(4):397–405. doi: 10.1093/dnares/dsu006 (PMC4131834; doi:10.1093/dnares/dsu006)
Supplement: Supplementary Data [file supp_21_4_397__index.html]

Construction of Pseudomolecule Sequences of the aus Rice Cultivar Kasalath for Comparative Genomics of Asian Cultivated Rice — Supplementary Data 

# Construction of Pseudomolecule Sequences of the *aus* Rice Cultivar Kasalath for Comparative Genomics of Asian Cultivated Rice

## Supplementary Data

Supplementary Data

**Files in this Data Supplement:**

- Supplementary Data - Supplementary Data
