# Supplementary material for: Construction of Pseudomolecule Sequences of the aus Rice Cultivar Kasalath for Comparative Genomics of Asian Cultivated Rice
Source: DNA Res. 2014 Feb 26;21(4):397–405. doi: 10.1093/dnares/dsu006 (PMC4131834; doi:10.1093/dnares/dsu006)

## Slide 1
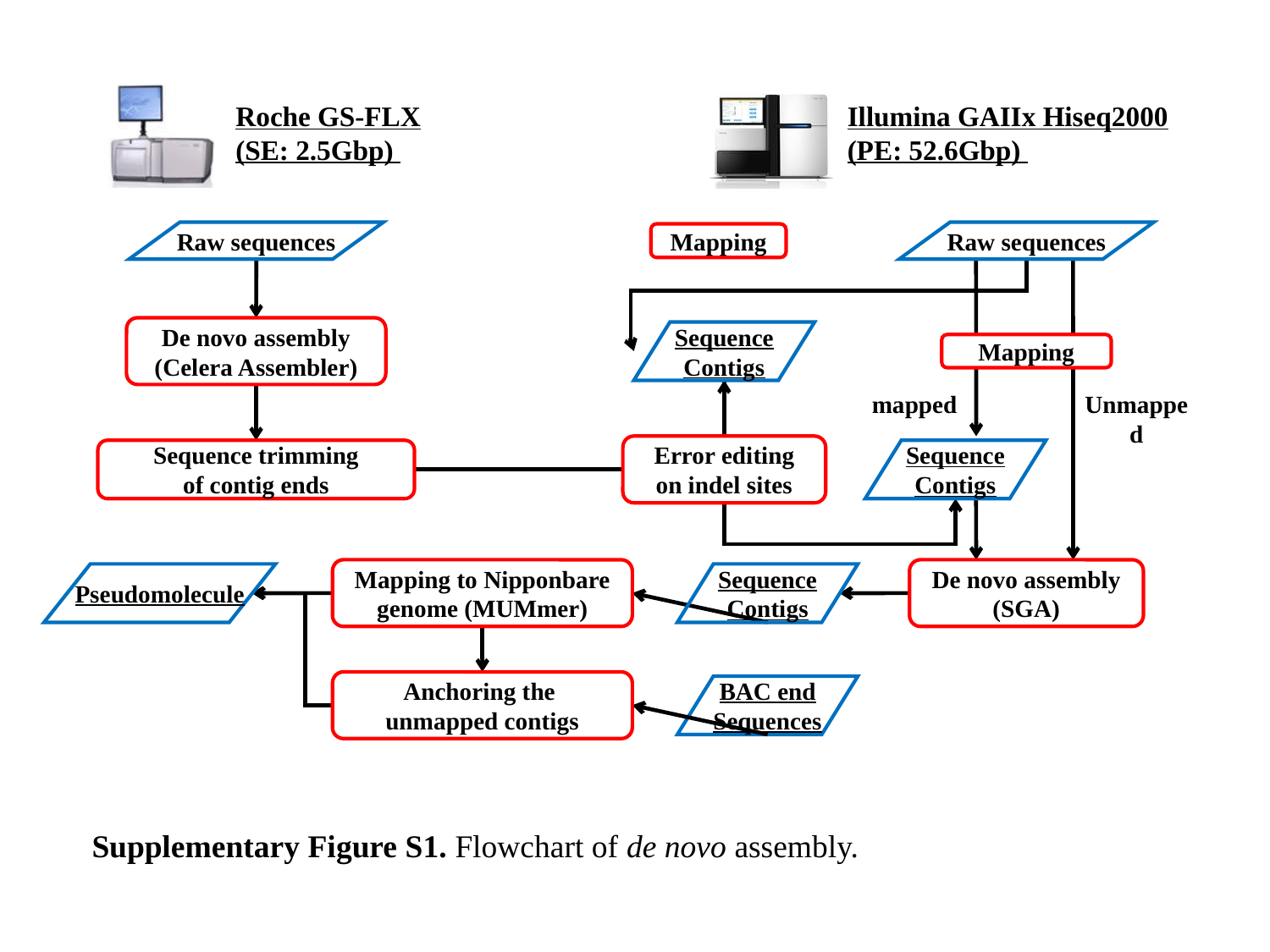

Roche GS-FLX
(SE: 2.5Gbp)
Illumina GAIIx Hiseq2000
(PE: 52.6Gbp)
Raw sequences
Raw sequences
Mapping
De novo assembly
(Celera Assembler)
Sequence
Contigs
Mapping
mapped
Unmapped
Error editing on indel sites
Sequence trimming
of contig ends
Sequence
Contigs
Mapping to Nipponbare genome (MUMmer)
De novo assembly
(SGA)
Pseudomolecule
Sequence
Contigs
Anchoring the
unmapped contigs
BAC end
Sequences
Supplementary Figure S1. Flowchart of de novo assembly.

## Slide 2
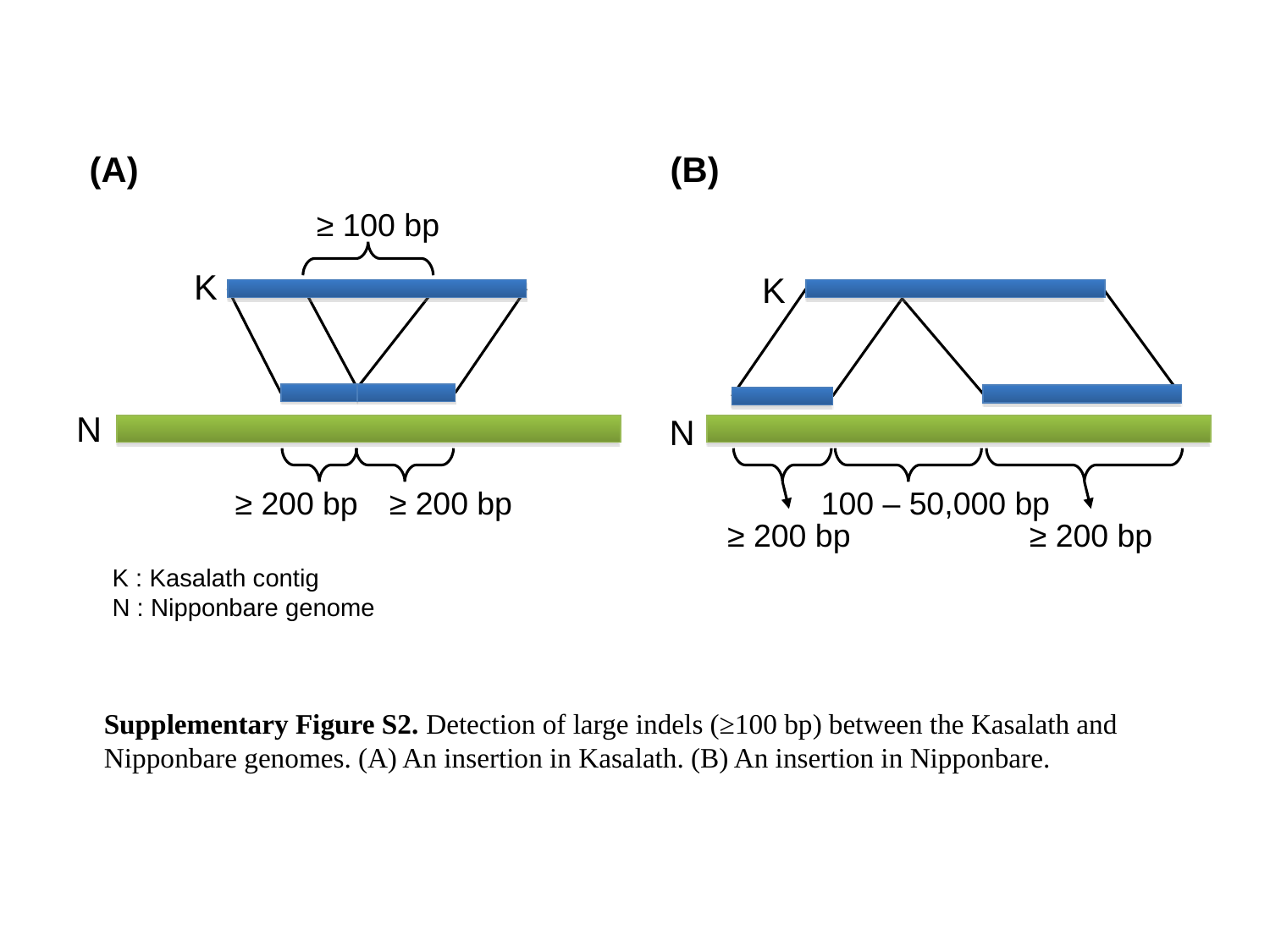

(A)
(B)
≥ 100 bp
K
K
N
N
≥ 200 bp
≥ 200 bp
100 – 50,000 bp
≥ 200 bp
≥ 200 bp
K : Kasalath contig
N : Nipponbare genome
Supplementary Figure S2. Detection of large indels (≥100 bp) between the Kasalath and Nipponbare genomes. (A) An insertion in Kasalath. (B) An insertion in Nipponbare.

## Slide 3
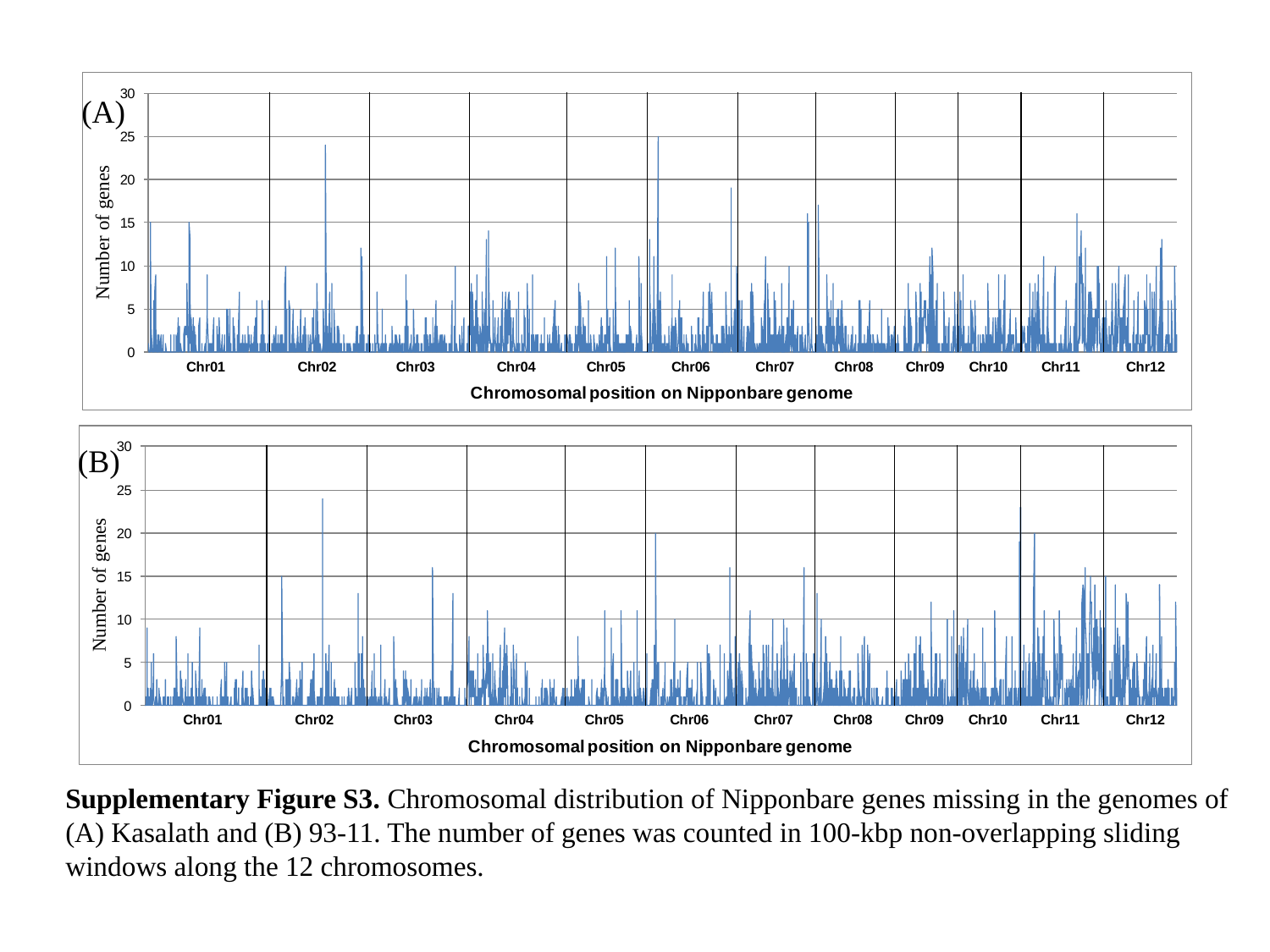

(A)
Number of genes
(B)
Number of genes
Supplementary Figure S3. Chromosomal distribution of Nipponbare genes missing in the genomes of (A) Kasalath and (B) 93-11. The number of genes was counted in 100-kbp non-overlapping sliding windows along the 12 chromosomes.

## Slide 4
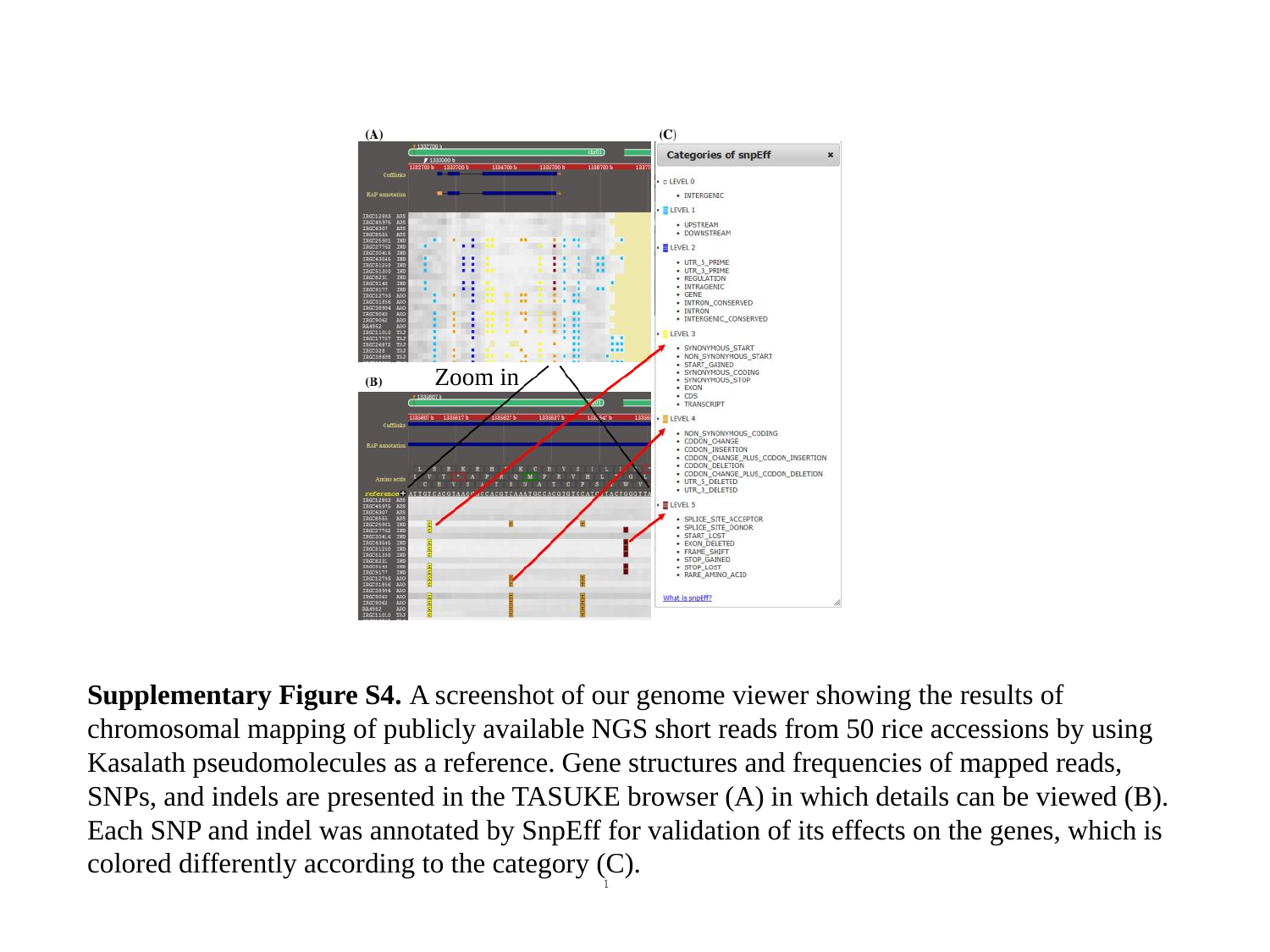

Zoom in
Supplementary Figure S4. A screenshot of our genome viewer showing the results of chromosomal mapping of publicly available NGS short reads from 50 rice accessions by using Kasalath pseudomolecules as a reference. Gene structures and frequencies of mapped reads, SNPs, and indels are presented in the TASUKE browser (A) in which details can be viewed (B). Each SNP and indel was annotated by SnpEff for validation of its effects on the genes, which is colored differently according to the category (C).

## Slide 5
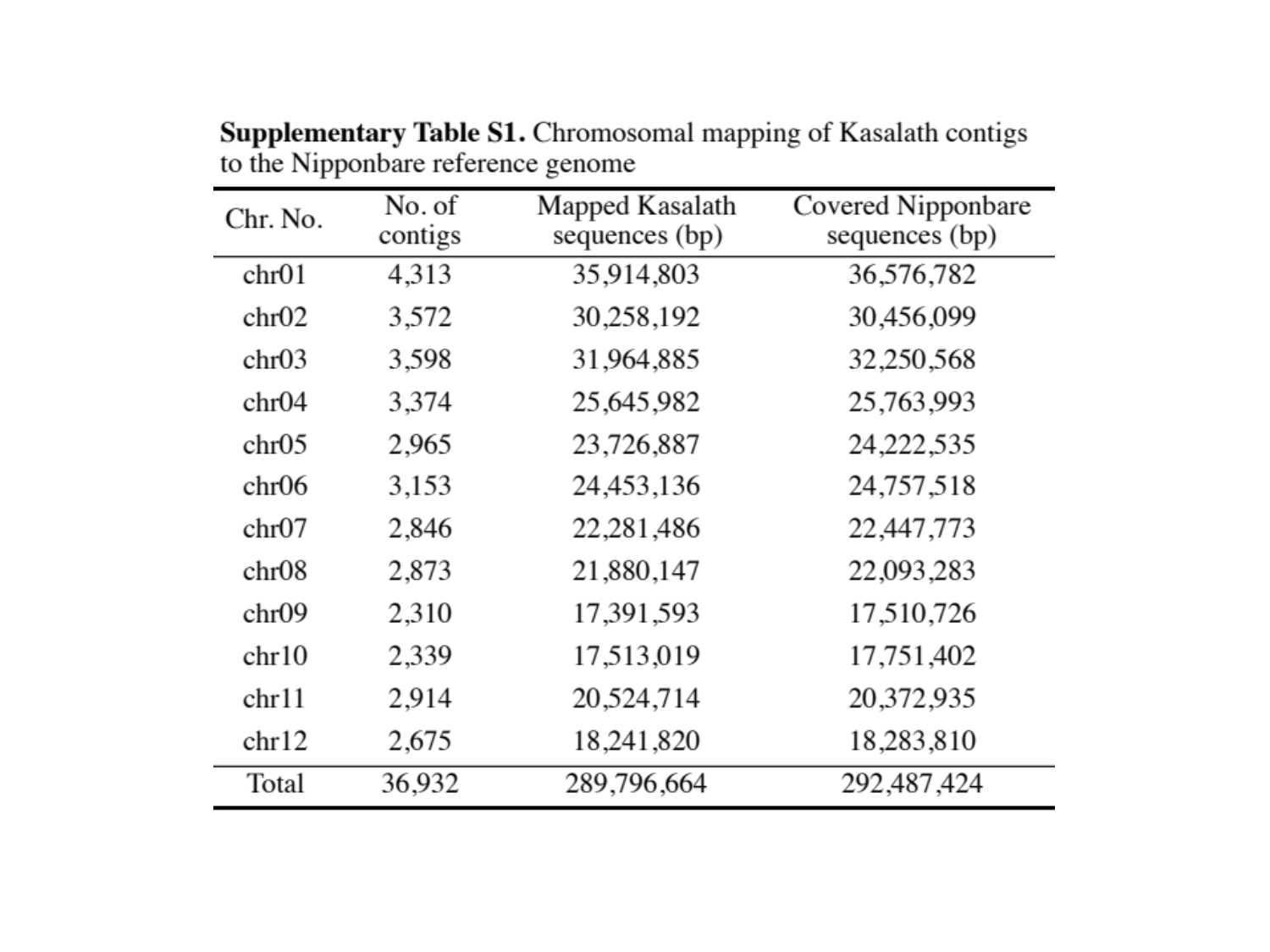

## Slide 6
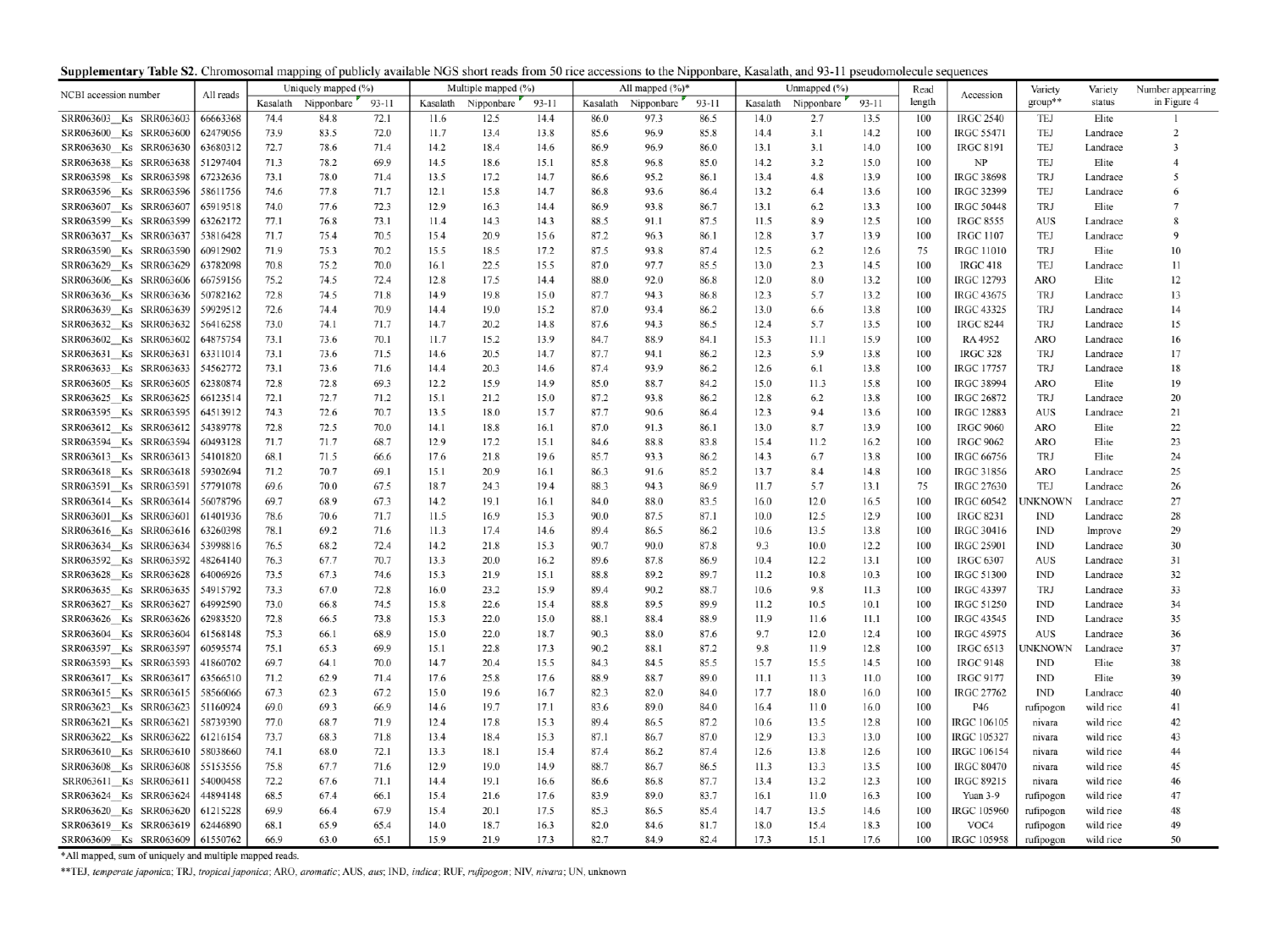

Supplement: Supplementary Data [file supp_dsu006_dsu006supp.ppt]
